# Arbuscular mycorrhizal fungal spore communities and co-occurrence networks demonstrate host-specific variation throughout the growing season

**DOI:** 10.1007/s00572-024-01168-2

**Published:** 2024-09-18

**Authors:** Jacob R. Hopkins, James D. Bever

**Affiliations:** 1 Evolution, Ecology, and Organismal Biology Department, The Ohio State University, 318 W 12th Avenue Aronoff Laboratory floor 3 Columbus, Columbus, OH 43201 USA; 2 Department of Ecology & Evolutionary Biology, University of Kansas, 1200 Sunnyside Avenue Lawrence, KS, 66045 USA; 3 Kansas Biological Survey, University of Kansas, 2101 Constant Ave. Lawrence, KS, 66047 USA

**Keywords:** Seasonality, Fungal communities, Niche partitioning, Mutualism, Grasslands

## Abstract

Microbial community assembly involves a series of ecological filtering mechanisms that determine the composition of microbial communities. While the importance of both broad and local level factors on microbial communities has been reasonably well studied, this work often is limited to single observations and neglects to consider how communities change over time (i.e., seasonal variation). Because seasonal variation is an important determinant of community assembly and determines the relative importance of community assembly filters, this represents a key knowledge gap. Due to their close associations with seasonal variation in plant growth and fitness, arbuscular mycorrhizal (AM) fungi are useful groups for assessing the importance of seasonal dynamics in microbial community assembly. We tested how seasonal variation (spring vs. summer), plant life history stage (vegetative vs. flowering), and host plant species (*Baptisia bracteata* var. leucophaea & *Andropogon gerardii*) influenced AM fungal spore community assembly. AM fungal spore community temporal dynamics were closely linked to plant host species and life history stage. While AM fungal spore communities demonstrated strong turnover between the spring (e.g., higher sporulation) and late summer (e.g., higher diversity), the strength and direction of these changes was modified by host plant species. Here we demonstrate the importance of considering temporal variation in microbial community assembly, and also show how plant-microbe interactions can modify seasonal trends in microbial community dynamics.

## Introduction

Microbial community assembly involves a series of ecological filters and processes that determine the composition of microbial communities (Morin 2011). Community assembly is first determined by broad scale factors like regional species pool, dispersal, and climate, followed by local level variation in abiotic and biotic conditions that influence the abundance of individual species (Vellend 2010; Kraft et al. 2015; Funk 2021). While the importance of broad (e.g., climate and dispersal; Barberán et al. 2014; Kivlin et al. 2011; Powell et al. 2015) and local level factors (e.g., soil conditions, disturbance, species interactions; Bahram et al. 2018; Fujita et al. 2023; Hopkins and Bennett 2023; Nemergut et al. 2013) on microbial community assembly has been reasonably well studied, this work often is limited to individual, static time points and does not consider the importance of seasonal variation (Shinohara et al. 2023; but see Lundberg et al. 2012; Shi et al. 2015; Aleklett et al. 2022). Because temporal variation is an important determinant of broad (e.g., seasons and successional stage; Bennett et al. 2013; Duhamel et al. 2019; Hopkins et al. 2021; Yang et al. 2020) and local level community assembly filters (e.g., nutrient availability and life history stage; Bahram et al. 2015; Hopkins et al. 2023; Mouquet et al. 2003), our poor understanding of seasonal variation represents a key gap in our understanding of microbial community assembly.

Many microbial taxa display seasonal trends in abundance (i.e., seasonality) that are directly related to seasonal variation in community assembly filters (Harvey et al. 1978; Santos-Gonzalez et al. 2007; Buckeridge et al. 2013). When measured, seasonality, or sampling time, is often the greatest determinant of microbial community composition, and outweighs local effects of nutrient availability and disturbance (Hopkins et al. 2023; Nemergut et al. 2013; Shinohara et al. 2023). Groups such as arbuscular mycorrhizal fungi (AM; obligate mutualists of > 80% of land plants) are key examples of this, as their sporulation and fitness is closely tied to seasonal changes in plant communities and plant growth (e.g., peaks in sporulation during and just after the plant growing season; (Smith and Read 2010; Deveautour et al. 2020b; Hopkins et al. 2024). Further, AM fungi also display species specific variation in abundance that can be tied to different host species (Bever et al. 1996; Eom et al. 2000; Kivlin et al. 2011) and changes in season (spring vs. summer; Pringle and Bever 2002; Santos-Gonzalez et al. 2007). Some of this variation is likely because of different growing periods of host plants (i.e., spring ephemerals vs. warm season grasses) that allow for seasonal niche differentiation amongst AM fungal symbionts (Su et al. 2011; Bennett et al. 2013). The close connections between AM fungi and their plant hosts demonstrates the importance of considering seasonal variation in community assembly.

Seasonal variation in AM fungal communities may further vary with the life history stage of the host plant (e.g., vegetative vs. flowering stage). Because the nutrient requirements of plant hosts change with life history stage (Chapin 1980; Römer and Schilling 1986; Grant et al. 2001), this could produce changes in the phosphorus (P) for carbon (C) exchange between host plants and AM fungal symbionts (Reynolds et al. 2006; Johnson et al. 2015; Ji and Bever 2016), with implications for fungal fitness and communities. For example, when plants are actively growing, greater amounts of P are required which could favor the fitness of AM fungal taxa that provide host plants with substantial P (Bever et al. 2009; Kiers et al. 2011). When plants flower or senesce for the year, however, plant nutrient demand is expected to decrease and correspondingly reduce C transfer to AM fungal symbionts (Lekberg et al. 2013). If changes in resource allocation alter the competitive ability of AM fungal symbionts, this could influence the interactions between species that influence community assembly (Bennett and Bever 2009; Christian and Bever 2018). AM fungal community assembly is also likely modified by host plant species, as plants vary in mycorrhizal responsiveness (i.e., the benefit a plant receives from association with AM fungi; Wilson and Hartnett 1998; Koziol and Bever 2015; Deveautour et al. 2021) and their ability to differentiate between more versus less beneficial AM fungal symbionts (Bever et al. 2009; Hopkins et al. 2023). This means that seasonal variation in AM fungal community assembly likely varies with not only season and plant life history stage, but also differences in plant-AM fungal interactions.

We tested how seasonal variation, plant life history stage (vegetative vs. flowering), and host plant species influenced AM fungal spore community assembly. We sampled AM fungal spore communities during the vegetive and flowering phases of two grassland species *Baptisia bracteata* var. *leucophaea* (which flowers in late spring-early summer and is vegetative in summer) and *Andropogon gerardii* (vegetative during spring-summer, flowers in late summer-early fall). This allowed us to test how plant life history stage and plant species influenced: (1) AM fungal spore community composition, (2) the abundances of individual AM fungal taxa, and (3) the associations between AM fungal taxa. We hypothesized that AM fungal spore community composition and species associations would shift between plant life history stages and seasons, with greater diversity and sporulation in the fall (when plants senesce) and lower community stochasticity during the flowering stage (early summer-*B. bracteata*; early fall- *A. gerardii*). We further hypothesized that seasonal variation in AM fungal spore community assembly and species associations would vary between plant hosts.

## Methods

### Study system

This work was conducted at the Anderson County Prairie Preserve (38° 10’ N, -95° 16’ W; Anderson County, KS). The preserve encompasses almost 1,400-acres that are maintained with annual to biennial fire, grazing, and mowing management. This work occurred in tract 13, which is a remnant tallgrass prairie. Soils at this site are part of the Clareson-Rock outcrop complex (USDA NRCS 2023). The site hosts a diverse spring and summer floral assemblage of forb, legume, and graminoid vegetation, including members of *Asclepias*, *Baptisia*, *Dalea*, *Andropogon*, *Helianthus*, *Liatris*, *Schizachyrium*, and *Amorpha* (Kansas Biological Survey 2010). Average annual temperatures range from 7 °C to 19 °C. Average annual precipitation is 970.3 mm with the majority occurring between April and September.

In this work, *Baptisia bracteata* var. *leucophaea* (C3 forb; plains wild-indigo) and *Andropogon gerardii* (C4 grass; big bluestem) were used as representative prairie plants. These taxa were chosen because of their relative dominance in tract 13 and their differences in seasonality. *B. bracteata* is an herbaceous perennial legume that emerges and is physiologically active in early spring and flowers in mid-spring. *A. gerardii* is a perennial, warm-season bunchgrass that emerges mid spring, is physiologically active in the heat of summer and flowers in the mid to late summer. Both plant species are responsive to AM fungi, with *(A) gerardii* growth nearly doubling when grown with AM fungi (mycorrhizal responsiveness = 99%) and *(B) bracteata* demonstrating an 83% increase in growth (Wilson and Hartnett 1998).

### Plot set-up

Experimental plots (*n* = 10) containing pairs of *B. bracteata* and *A. gerardii* (plants within each pair separated by ≤ 1 m) were established in spring 2019. Plots were marked with plastic marker flags for easy rediscovery at each sample time. Because of varied fire history at tract 13 (half of the tract burned in October 2018), fire history was recorded for each plot to account for variation in management.

### Field sampling

AM fungal spore community samples were collected in June 2019 (end of *B. bracteata* flowering) and in September 2019 (end of *(A) gerardii* flowering). AM fungal spore communities were used for assessment of community composition because they allow for assessment of viability, sorting into morphospecies, are directly indicative of fitness (Bever et al. 1996; Bever 2002), are closely linked to plant community dynamics (Su et al. 2011; Middleton and Bever 2012), and are reliable indicators of seasonal variation in belowground communities (Pringle and Bever 2002). A 2 cm diameter soil corer was used to collect a single rhizosphere sample (depth of 15 cm) next to the base of each *(B) bracteata* (*n* = 10) and *A. gerardii* plant (*n* = 10) at each sampling time (*n* = 2). This produced 20 AM fungal spore samples for each sampling time, for a total of 40 samples across the entire study period. Samples were kept cool with ice packs in the field and then stored at 4 °C within six hours of collection. The soil corer was cleaned with paper towels and sterilized with 70% EtOH between samples.

AM fungal spores were extracted from soil samples within 2–4 weeks of collection using 2 mm and 38 μm sieves, followed by centrifugation with 60% sucrose solution. Extracted spores were stored in water at 4 °C until communities were quantified.

### Spore community analysis

AM fungal spore communities were quantified using a Nikon dissection scope (Nikon, Tokyo, Japan) at 30x magnification. Spores were sorted into morphotypes based on pigmentation color, size, internal lipid contents, and hyaline appearance. Counts for each morphotype, total spore count (i.e., sporulation), and diversity (inverse Simpson metric) were recorded for each sample. When possible, putative classifications were applied to morphotypes using INVAM species descriptions (INVAM 2022).

### Statistical analyses

All analyses were conducted in R version 4.3.2 (R Core Team 2022). We tested how plant host ID (*B. bracteata* and *A. gerardii*) and sampling time (spring vs. summer) influenced AM fungal spore community composition using principle coordinates analyses (PCoA) and permutational multivariate analysis of variance (PERMANOVA) with the Vegan package (Oksanen et al. 2013). Bray-Curtis dissimilarity matrices and ordinations were produced for AM fungal spore communities using the vegdist() and prcomp() functions. Following ordination, a PERMANOVA was used to test the effect of plant host ID, sampling time, and their interaction effects on AM fungal spore communities using the adonis2() function. The PERMANOVA model also accounted for prior fire history and location effects. The fire history term (i.e., presence/absence of Fall 2018 fires) was included first to account for its effect because the adonis function uses sequential sums of squares. To account for plot level variation, permutations (*n* = 999) were restricted to within sampling plot.

Plant host ID and sampling time effects on AM fungal spore community diversity (continuous; Inv. Simpson), community beta dispersion (continuous; Bray-Curtis), sporulation (count), and morphotype abundance (count) were assessed using either type III linear mixed effect (LMERs; continuous data) or type III generalized linear mixed effect models (GLMERs; poisson link function, count data) using either the lmer() or glmer() functions (lme4 package; Bates et al. 2015) followed by the joint_tests() function (emmeans package; Lenth 2018). LMER and GLMER models included plant host ID, sampling time, and their interaction as fixed effects, and controlled for plot and fire history. Note that the interaction term represents spore community turnover between plant host life history stages (e.g., Spring growth vs. Summer flowering times in *A. gerardii*). Following significant main effects, estimated marginal means were extracted using the emmeans() function and tested with contrasts using the contrast() function. Due to rarity and low sporulation, it was not possible to test changes in abundance for every spore morphotype.

Plant host ID and sampling time effects on intra-community associations were tested using network analysis tools available in the NetCoMi package (Peschel et al. 2021). AM fungal spore community networks were first constructed for plant host species at each sampling time using the netConstruct() function. This allowed for comparison of AM fungal spore community co-occurrence networks between (e.g., summer *B. bracteata* – flowering stage vs. fall *B. bracteata* – vegetative stage) and within (e.g., summer *B. bracteata* vs. summer *A. gerardii*) sampling times. Networks were created using a matched-pairs design (controls for plot effects), biweight midcorrelation association functions (robust to outliers), and a sparsification threshold of 0.3. Network metrics (Table 1) for the largest connected component (LCC) and entire network were measured using the netAnalyze() function with the “cluster_fast_greedy” clustering algorithm. Hub taxa were identified using combinations of node degree, betweenness, closeness, and eigen vector with hub threshold set to 0.9 (combined values must exceed 0.9 to be considered a hub taxon). Network metrics were then compared using the netCompare() function with permutations set to 1000.

**Table 1:** Description of network analysis metrics

Metric	Applies to	Description
Relative LCC size	LCC	size comparison of largest connected component
Clustering coefficient	LCC, Whole	degree to which nodes cluster together (1 = all connected, 0 = not connected)
Modularity	LCC, Whole	degree to which network divides into sub-groups
Positive edge percentage	LCC, Whole	percentage of positive edges in a network
Edge density	LCC, Whole	ratio of observed vs. possible edges
Natural connectivity	LCC, Whole	how resistant network is to removing edges (higher value = more robust)
Vertex connectivity	LCC	smallest number of vertices whose deletions causes graph to not be connected
Edge connectivity	LCC	smallest number of edges whose deletion causes graph to not be connected
Average dissimilarity	LCC, Whole	measure of path coefficients (higher values mean lower coefficients)
Average path length	LCC	average no. of steps to get from one node to another
Graphlet correlation dist.	LCC, Whole	compares similarity between two networks
Number of components	Whole	number of sub-graphs in a network

## Results

### Plant host ID and sampling time determine AM fungal spore community composition

**Fig. 1:**
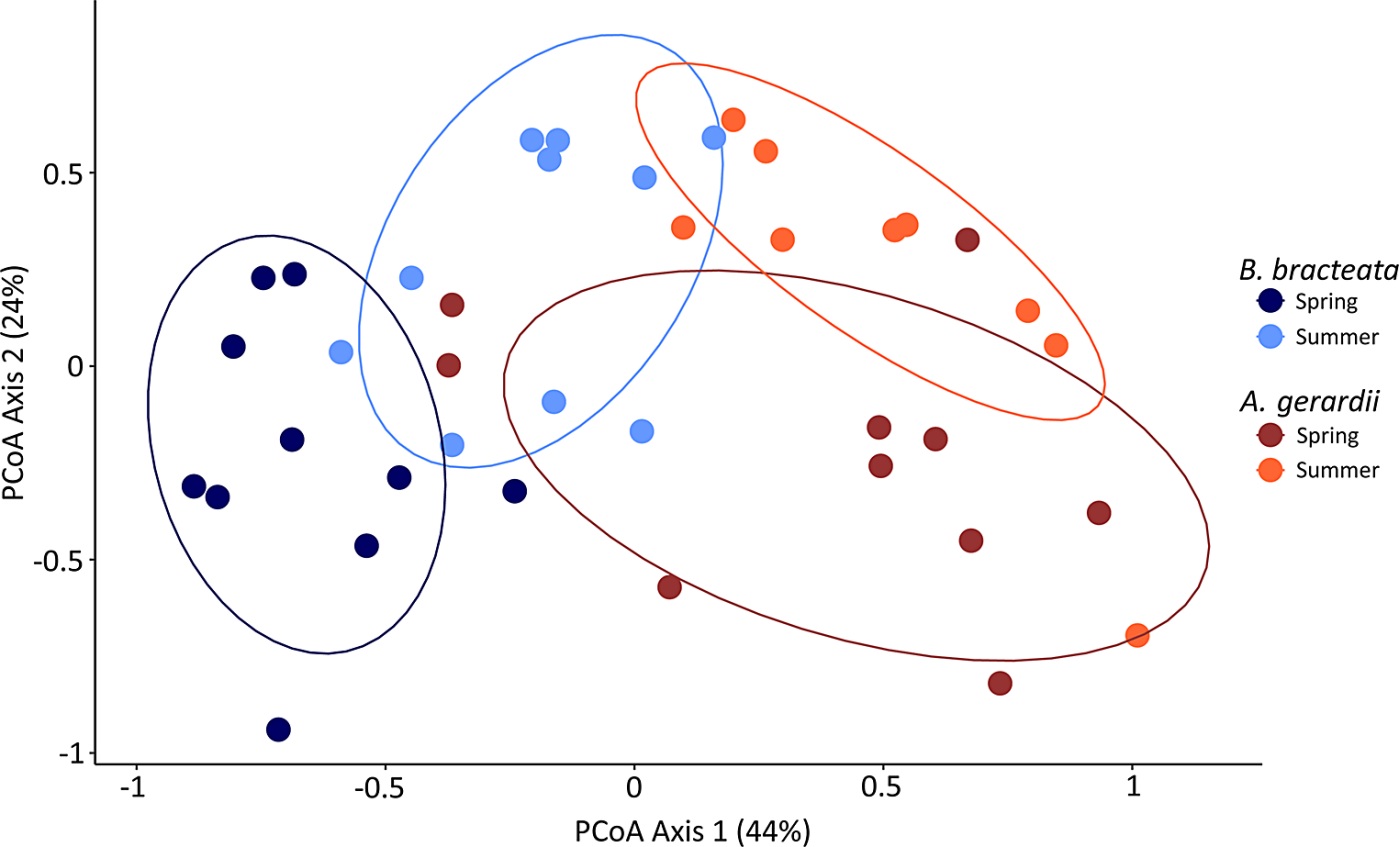
AM fungal spore community PCoA. AM fungal spore community composition differed between plant hosts and demonstrated seasonal turnover in composition. Ellipses denote 75% confidence intervals

**Fig. 2:**
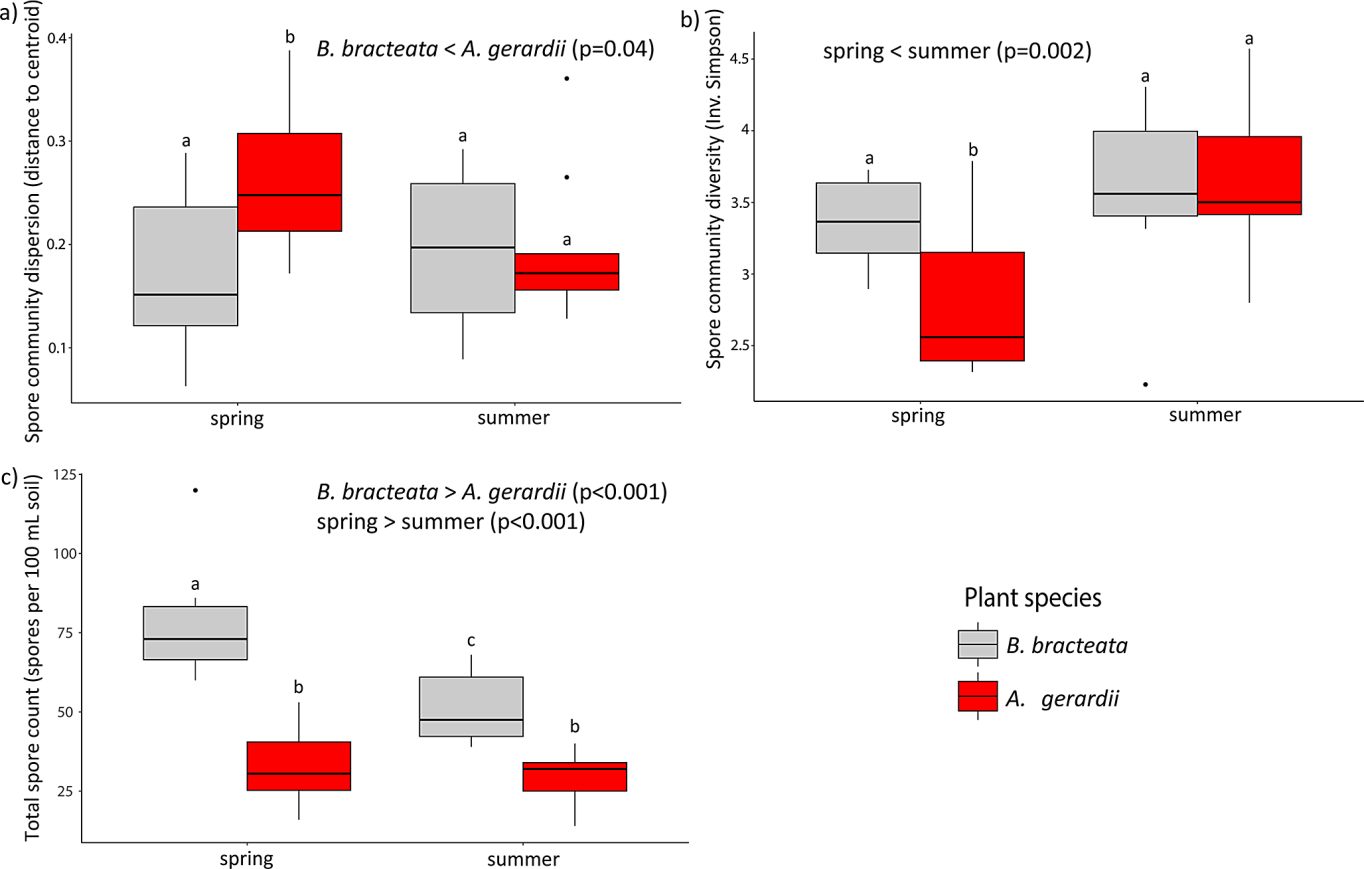
AM fungal spore community (**a**) beta dispersion, (**b**) spore morphotype diversity, and (**c**) total sporulation responses to season and plant host species. Whiskers extend from the upper and lower quartiles to largest/lowest value no further than 1.5 times the inter-quartile range. Different lower case letters denote significant differences (*p* < 0.05) between treatment groups

*B. bracteata* and *(A) gerardii* were associated with distinct AM fungal spore communities (F_1,34_=15.3, *p* = 0.001, R^2^ = 0.26; Table 2; Fig. 1) that displayed significant seasonal turnover between the spring and summer (F_1,34_=8.04, *p* = 0.001, R^2^ = 0.14). In total, 12 different AM fungal species were identified, with 8 taxa common to both host plant species, 1 taxon found only in *(B) bracteata*, 2 taxa unique to *(A) gerardii*, 3 taxa found only in spring, and 2 taxa found only in the summer. *(B) bracteata* spore communities were less stochastic (lower beta-dispersion; F_1,26.3_=4.8, *p* = 0.04; Table 3; Fig. 2a), were marginally more diverse (F_1,26.3_=3.9, *p* = 0.06; Fig. 2b), and had higher sporulation (F_1,Inf_=198, *p* < 0.001; Fig. 2c) than *(A) gerardii* spore communities. Further, *(B) bracteata* spore communities demonstrated lower sporulation during the summer sampling time (vegetative phase; *p* < 0.001). *A. gerardii* spore communities became less stochastic (lower beta-dispersion; *p* = 0.04) and more diverse (*p* < 0.001) during the summer sampling time (flowering phase). In summary, plant host ID was the strongest determinant of AM fungal spore community composition; however, spore communities associated with each plant host species displayed significant seasonal variation.

**Table 2:** AM fungal spore community PERMANOVA table

Model term	D.F.	Sum Sqrs.	*R* ^2^	F	*p*-value
Plant species	1	0.85	0.26	15.3	0.001*
Season	1	0.45	0.14	8.04	0.001*
Species x season	1	0.07	0.02	1.3	0.27
Fire history	1	0.005	0.002	0.09	0.001*
Residual	34	1.9	0.58		

*: *p* < 0.001

**Table 3:** AM fungal community beta dispersion linear mixed effect model and total sporulation and morphotype diversity generalized linear mixed effect model tables

Model term	Beta-dispersion					Total sporulation					Spore morphotype diversity				
	*D.F.1*	*D.F.2*	*F*	*P*		*D.F.1*	*D.F.2*	*F*	*P*		*D.F.1*	*D.F.2*	*F*	*P*	
*Plant species*	1	26.3	4.78	0.04*		1	Inf	197.5	0.001***		1	26.3	3.9	0.06 .	
*Season*	1	26.3	1.1	0.3		1	Inf	26.3	0.001***		1	26.3	12.4	0.002*	
*Species x season*	1	26.3	4.3	0.05*		1	Inf	8.9	0.003*		1	26.3	4.1	0.05*	
*Random effects*	*Var.*	*Std. Dev.*				*Var.*	*Std. Dev.*				*Var.*	*Std. Dev.*			
*Plot*	0.0006	0.02				0.02	0.1				0.02	0.1			
*Fire history*	0	0				0	0				0	0			
*Contrast*	*Estim.*	*S.E.*	*d.f.*	*T*	*P*	*Estim.*	*S.E.*	*d.f.*	*T*	*P*	*Estim.*	*S.E.*	*d.f.*	*T*	*P-value*
*B. bracteata vs. A. gerardii*	-0.1	0.045	26.3	-2.17	0.04*	4.1	0.4	Inf	14.1	0.001***	0.6	0.3	26.3	14.1	0.06 .
*Spring vs. Fall*	-0.05	0.045	26.3	1.05	0.3	0.6	0.06	Inf	-5.1	0.001***	-1	0.3	26.3	-5.1	0.002**
*B. bracteata: Spring vs. Summer*	-0.02	0.031	26	-0.74	0.5	0.7	0.04	Inf	-7.2	0.001***	0.2	0.2	26	-7.2	0.29
*A. gerardii: Spring vs. Summer*	0.07	0.032	26.6	2.17	0.04*	0.9	0.07	Inf	-1.3	0.2	-0.8	0.2	26.5	-1.3	0.001***

*p* < 0.1; *: *p* < 0.05; **: *p* < 0.01; *** *p* < 0.001

### Plant host ID and sampling time influence species abundances

**Fig. 3:**
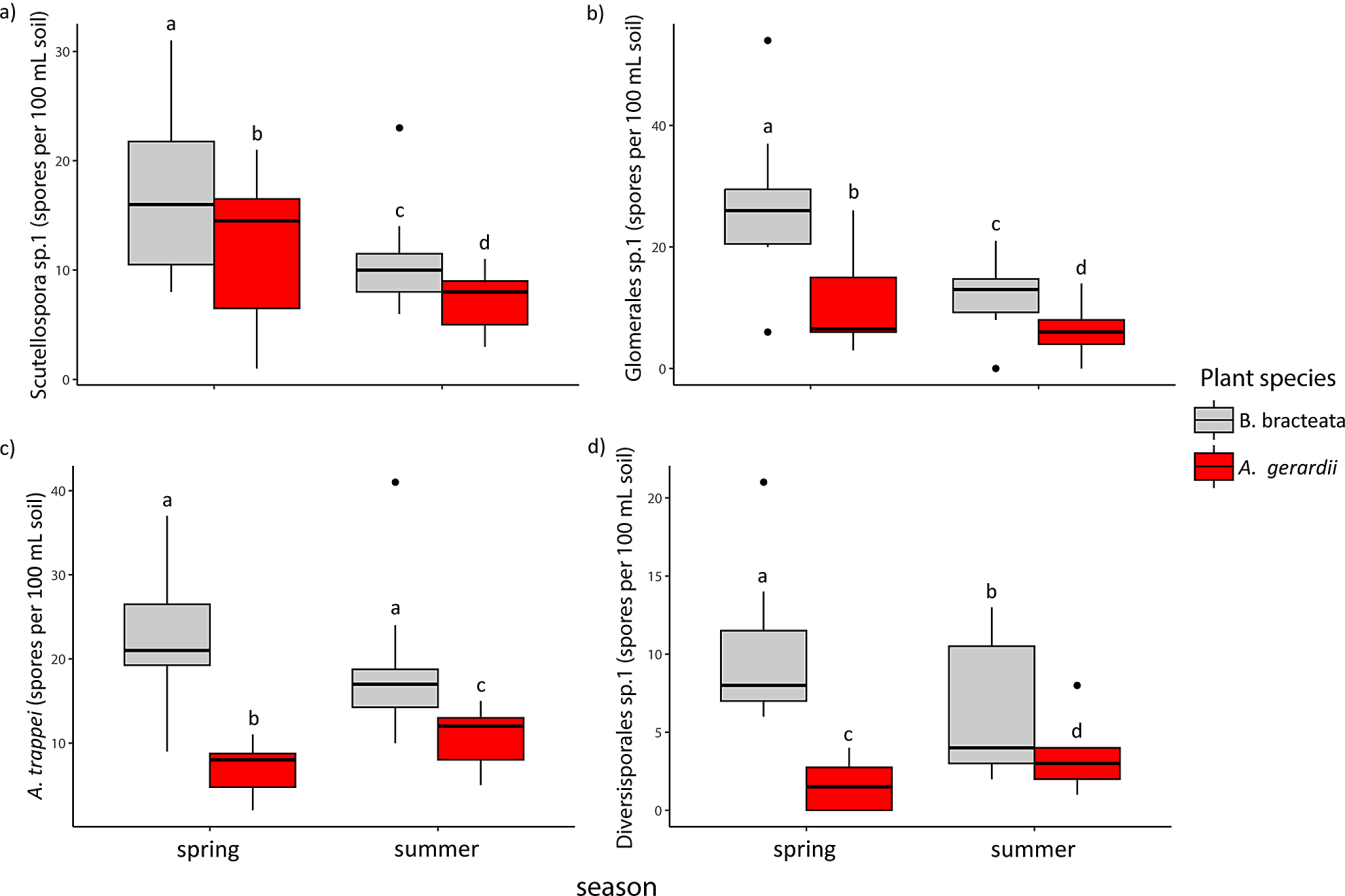
AM fungal spore abundance responses to season and plant host. Means followed by the same letter do not differ significantly by t-tests on estimated marginal means (*p* < 0.05) between treatment groups

The abundances of AM fungal species differed with sampling time and between plant hosts. During the spring sampling time, *Scutellospora* sp.1 (F_1,Inf_=24, *p* < 0.001; Table 4; Fig. 3a) and Glomerales sp.2 (F_1,Inf_=51, *p* < 0.001, Fig. 3b) abundances were highest relative to the summer. Further, *B. bracteata* plants hosted greater abundances of *Archaeospora trappei* (F_1,Inf_=85, *p* < 0.001, Fig. 3c), Diversisporales sp.1 (F_1,Inf_=48, *p* < 0.001, Fig. 3d), *Scutellospora* sp. 1 (F_1,Inf_=16, *p* < 0.001), and *Glomerales* sp.2 (F_1,Inf_=64, *p* < 0.001) relative to *A. gerardii* hosts. Changes in seasonal species abundances also displayed host-specific patterns. Specifically, *(A) trappei* (F_1,Inf_=8.6, *p* = 0.003) and Diversisporales sp.1 (F_1,Inf_=12, *p* < 0.001) abundances decreased during the summer with *(B) bracteata* hosts, but increased during the summer with *A. gerardii* hosts. To conclude, AM fungal species demonstrated seasonal variation in abundance that was modified by plant host species.

**Table 4:** AM fungal spore morphotype abundance generalized linear mixed effect model tables

Model term			*A. trappei*		Diversisporales sp.1		*Scutellospora* sp. 1		Glomerales sp.1		Glomerales sp.2		*Gigaspora* sp.1	
	D.F.1	D.F.2	F	*P*	F	*P*	F	*P*	F	*P*	F	*P*	F	*P*
Plant species	1	Inf	84.8	0.001***	48.1	0.001***	15.9	0.001***	0	1	64.1	0.001***	0.04	0.8
Season	1	Inf	1.9	0.2	0.5	0.5	23.8	0.001***	0	1	50.7	0.001***	0.08	0.8
Species x season	1	Inf	8.6	0.003**	12.1	0.001***	0.01	0.9	0.001	1	1.2	0.3	1	0.3
Random effects			Var.	Std. Dev.	Var.	Std. Dev.	Var.	Std. Dev.	Var.	Std. Dev.	Var.	Std. Dev.	Var.	Std. Dev.
Plot			0.02	0.1	0	0	0.06	0.3	3.8	1.9	0.1	0.3	0.7	0.8
Fire history			0	0	0	0	0	0	2.1	1.5	0	0	0	0
*Contrast*			Estim.	*P*	Estim.	*P*	Estim.	*P*	Estim.	*P*	Estim.	*P*	Estim.	*P*
*B. bracteata* vs. *A. gerardii*			1.7	0.001***	2.4	0.001***	0.79	0.001***	-	-	1.6	0.001***	-	-
Spring vs. Summer			0.26	0.16	0.25	0.5	0.96	0.001***	-	-	1.4	0.001***	-	-
*B. bracteata:* Spring vs. Summer			0.14	0.15	0.48	0.003**	0.47	0.001***	-	-	0.82	0.001***	-	-
*A. gerardii:* Spring vs. Summer			-0.4	0.01**	-0.73	0.02*	0.49	0.002**	-	-	0.6	0.001***	-	-

*: *p* < 0.05; **: *p* < 0.01; *** *p* < 0.001

### Plant host ID and sampling time structure AM fungal spore community networks

**Fig. 4:**
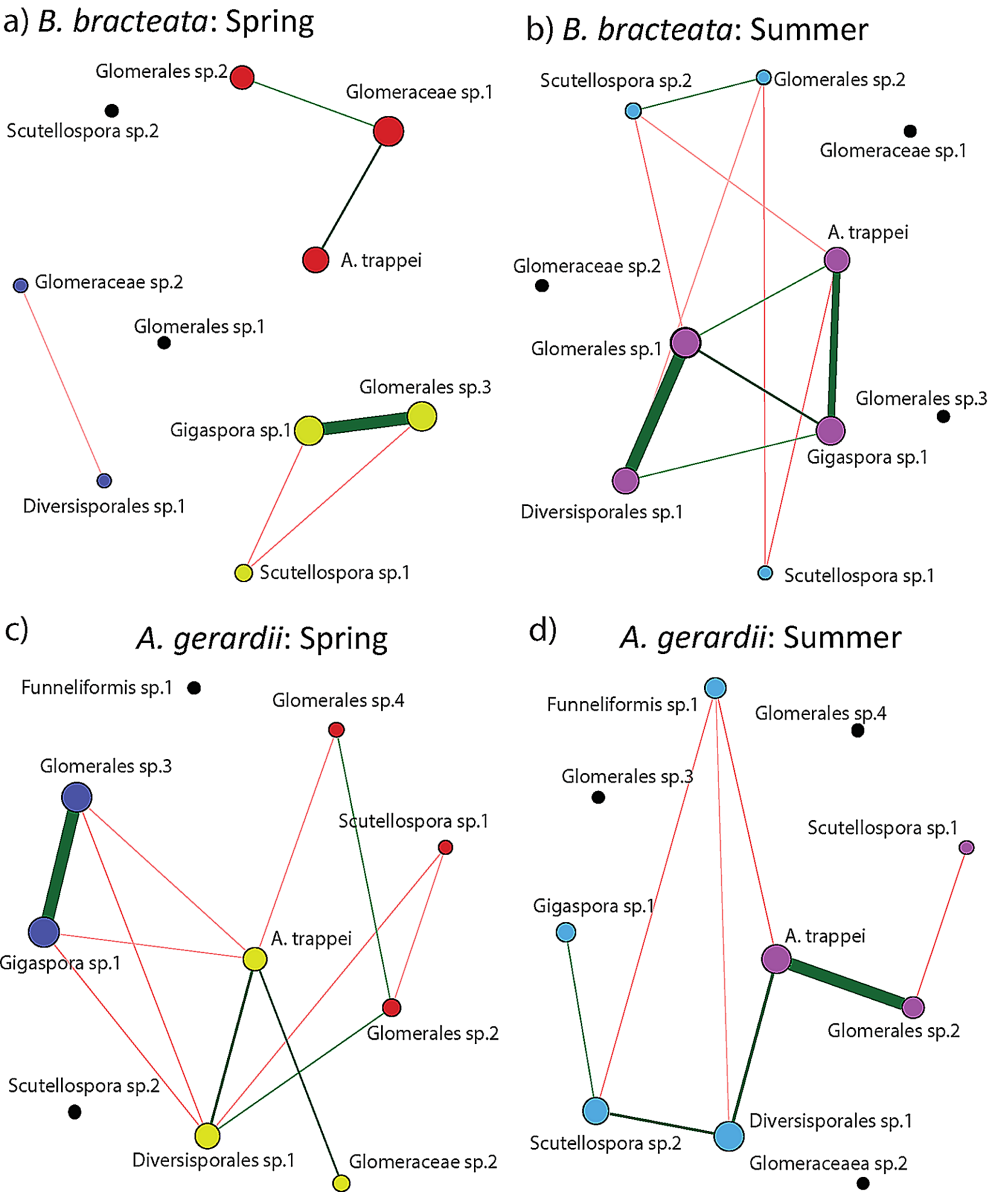
*B. bracteata* and *A. gerardii* AM fungal spore networks. Circles denote AM fungal spore taxa, edges denote significant correlations between AM fungal taxa (green = positive, red = negative), circle colors denote network groups. Line thickness denotes correlation strength, node sizes represent the eigenvector coefficient (larger size implies greater influence in the network)

AM fungal spore community network structure varied between sampling times and this effect was influenced by plant host species. *B. bracteata* networks displayed substantial changes in structure between the spring and summer. In the spring, the largest connected component (LCC) for *B. bracteata* was smaller (*p* = 0.03; Table 5; Fig. 4a and b), displayed a higher degree of clustering (*p* = 0.01), was less modular (*p* = 0.01), had a greater edge density (*p* = 0.004), greater natural connectivity (*p* = 0.002), and different topography (*p* = 0.01) than in the summer. At the whole network scale, modularity was higher in summer (*p* = 0.01), and network topography (i.e., graphlet correlation distance; *p* = 0.04) differed between the spring and summer. *A. gerardii* networks, however, did not vary in topography or structure between the spring and summer (Table 6; Fig. 4c and d).

**Table 5:** *B. Bracteata* Spring and Summer network comparison table

Metric	spring	summer	Abs. Diff.	*p*-value	
Largest connected component (LCC):					
Relative LCC size	0.25	0.58	0.33	0.03*	
Clustering coefficient	1	0.316	0.68	0.01**	
Modularity	-0.22	0.099	0.32	0.01**	
Positive edge percentage	33.3	54.545	21.2	0.2	
Edge density	1	0.52	0.48	0.004**	
Natural connectivity	0.7	0.26	0.44	0.002**	
Vertex connectivity	2	2	0	1	
Edge connectivity	2	2	0	1	
Average dissimilarity	0.56	0.64	0.08	0.2	
Average path length	0.56	0.95	0.39	0.2	
Graphlet correlation dist.	8.12			0.01**	
Whole network:					
Number of components	7	6	1	0.8	
Clustering coefficient	0.75	0.316	0.434	0.09	
Modularity	0.611	0.099	0.512	0.002**	
Positive edge percentage	50	54.545	4.545	0.8	
Edge density	0.091	0.167	0.076	0.4	
Natural connectivity	0.119	0.124	0.006	0.7	
Graphlet correlation dist.	4.03			0.04*	

.: *p* < 0.1, *: *p* < 0.05, **: *p* < 0.01

**Table 6:** *A. gerardii* network spring and summer network comparison table

Metric	Summer	Fall	Abs. diff.	*p*-value
Largest connected component (LCC):				
Relative LCC size	0.67	0.58	0.08	0.8
Clustering coefficient	0.57	0.35	0.22	0.4
Modularity	0.06	0.22	0.16	0.2
Positive edge percentage	41.7	50	8.33	0.7
Edge density	0.43	0.38	0.05	0.7
Natural connectivity	0.21	0.23	0.02	0.8
Vertex connectivity	1	1	0	1
Edge connectivity	1	1	0	1
Average dissimilarity	0.68	0.69	0.001	0.9
Average path length	1.12	1.3	0.19	0.4
Graphlet correlation dist.	4.46			0.3
Whole network:				
Number of components	5	6	1	0.8
Clustering coefficient	0.57	0.35	0.22	0.4
Modularity	0.06	0.22	0.16	0.2
Positive edge percentage	41.7	50	8.33	0.7
Edge density	0.18	0.12	0.06	0.5
Natural connectivity	0.12	0.11	0.001	0.4
Graphlet correlation dist.	2.97			0.4

Network structure also varied between *B. bracteata* and *(A) gerardii*; however, this effect was largely restricted to the summer. Spring *(B) bracteata* LCC’s were smaller (*p* = 0.03; Tables 7 and 8), had higher edge densities (*p* = 0.008), greater natural connectivity (*p* = 0.008), smaller path lengths (*p* = 0.03), and different topographies (*p* = 0.02) than *(A) gerardii* networks. At the whole network scale, spring *(B) bracteata* networks were more modular (*p* = 0.006) and had different topographies (*p* = 0.01) than *A. gerardii* networks. In the summer, however, network topography and structure did not differ between these plant hosts, with the exception of greater edge densities in *A. bracteata* LCC’s (*p* = 0.04). To summarize, AM fungal spore community network structure varied between the spring and summer, however, this effect was influenced by plant host ID.

**Table 7:** Spring *B. bracteata* and *A. gerardii* network comparison table

Metric	*B. bracteata*	*A. gerardii*	Abs. Diff.	*p*-value
Largest connected component (LCC):				
Relative LCC size	0.25	0.67	0.42	0.03*
Clustering coefficient	1	0.57	0.43	0.08 .
Modularity	-0.22	0.06	0.28	0.07 .
Positive edge percentage	33.3	41.7	8.33	0.8
Edge density	1	0.43	0.57	0.008**
Natural connectivity	0.7	0.21	0.49	0.008**
Vertex connectivity	2	1	1	0.4
Edge connectivity	2	1	1	0.4
Average dissimilarity	0.56	0.68	0.11	0.2
Average path length	0.56	1.12	0.55	0.03*
Graphlet correlation dist.	8.46			0.02*
Whole network:				
Number of components	7	5	2	0.47
Clustering coefficient	1	0.57	0.43	0.07 .
Modularity	0.61	0.06	0.55	0.006**
Positive edge percentage	57.1	41.7	15.5	0.6
Edge density	0.11	0.18	0.08	0.5
Natural connectivity	0.12	0.12	0.001	1
Graphlet correlation dist.	5.22			0.01**

.: *p* < 0.1, *: *p* < 0.05, **: *p* < 0.01

**Table 8:** Summer *B. bracteata* and *A. gerardii* network comparison table

Metric	*B. bracteata*	*A. gerardii*	Abs. Diff.	*p*-value
Largest connected component (LCC):				
Relative LCC size	0.5	0.58	0.08	0.6
Clustering coefficient	0.53	0.35	0.48	0.3
Modularity	0.03	0.22	0.19	0.2
Positive edge percentage	63.6	50	13.6	0.7
Edge density	0.73	0.38	0.35	0.04*
Natural connectivity	0.36	0.23	0.13	0.2
Vertex connectivity	2	1	1	0.4
Edge connectivity	2	1	1	0.4
Average dissimilarity	0.58	0.69	0.1	0.5
Average path length	0.77	1.3	0.53	0.1
Graphlet correlation dist.	3.7			0.7
Whole network:				
Number of components	7	6	1	0.8
Clustering coefficient	0.83	0.35	0.48	0.3
Modularity	0.03	0.22	0.19	0.2
Positive edge percentage	63.6	50	13.6	0.7
Edge density	0.17	0.12	0.05	0.6
Natural connectivity	0.14	0.11	0.02	0.4
Graphlet correlation dist.	2.16			0.8

.: *p* < 0.1, *: *p* < 0.05, **: *p* < 0.01

## Discussion

AM fungal spore community seasonal dynamics were closely linked to plant host species and life history stage. While AM fungal communities demonstrated strong turnover between the spring (e.g., higher sporulation) and late summer (e.g., higher diversity), the strength of these changes was modified by host plant species. *B. bracteata* generally hosted a larger, more diverse spore community than *A. gerardii*, however, the abundances of two AM fungal species were linked to host plant flowering times. Specifically, *A. trappei* (*A. gerardii*) and Diversisporales sp.1 (*B. bracteata* and *(A) gerardii*) abundances were highest during host flowering periods. Furthermore, AM fungal species associations also varied between seasons and plant hosts, with *(B) bracteata* associated AM fungal networks becoming less modular and less clustered in the summer versus the spring, and *A. gerardii* associated networks remaining relatively stable between seasons and life history stages. This builds on past work identifying the importance of seasonal variation (Pringle and Bever 2002; Santos-Gonzalez et al. 2007; Bennett et al. 2013; Deveautour et al. 2020b) and plant host species (Bever et al. 1996; Eom et al. 2000; Koziol and Bever 2015) on AM fungal community assembly by demonstrating how AM fungal seasonal community dynamics both vary between plant hosts and can be linked to plant life history stages. Because AM fungal community seasonal dynamics are closely linked to plant host ID and life history stage, it is critical that soil microbial ecologists consider both sampling time and host-plant life history stage when assessing microbial community assembly.

Seasonal variation in AM fungal community assembly was closely linked to host plant species and life history stage. AM fungal community composition remained distinct between *B. bracteata* and *(A) gerardii* throughout the growing season, with *(B) bracteata* hosting a larger (greater sporulation) and more diverse AM fungal symbiont community than *(A) gerardii*. Some of these differences are likely due to the leguminous nature of *(B) bracteata* (plants are less N limited), which can favor plant resource allocation to AM fungal symbionts, reduce competition among AM fungal taxa, and potentially explain the higher levels of sporulation and diversity (Bennett and Bever 2009; Johnson et al. 2010, 2015). While AM fungal spore community diversity was generally lower in *(A) gerardii* hosts relative to *(B) bracteata*, it is worth noting that diversity did increase when *(A) gerardii* flowered, whereas no change in diversity was observed for *(B) bracteata*. This may reflect shifts in *A. gerardii* nutrient requirements during the flowering stage (e.g., lower N and P requirements; Chapin 1980; Grant et al. 2001) and corresponding reductions in plant C allocation to AM fungal symbionts (Smith and Read 2010). If preferential allocation of plant C to specific AM fungal symbionts is reduced during *A. gerardii* flowering stages, this could alter the competitive abilities of AM fungal taxa and allow for increased diversity in AM fungal communities (Bennett and Bever 2009; Bever et al. 2009; Kiers et al. 2011; Bever 2015; Christian and Bever 2018; Hopkins et al. 2023a). The differences in AM fungal diversity and sporulation between the two host plant species demonstrates the importance of the host plant in the seasonal dynamics of AM fungal community assembly.

Seasonal variation in AM fungal community networks differed between plant host species. *B. bracteata* associated networks became less modular and largest connected component size (LCC) increased during the vegetative (summer) versus the flowering stage (spring). The higher level of between-species associations (lower modularity and larger LCCs) during *B. bracteata’s* vegetative stage would correspond with less plant physiological activity (lower photosynthate production) and greater competition for plant C among AM fungi (Johnson et al. 2010, 2015). Conversely, *A. gerardii* associated network structure did not change between vegetative (spring) and flowering stages (summer) despite subsequent changes in community composition, diversity, and sporulation. This implies that *A. gerardii* hosts a relatively stable AM fungal network throughout the growing season with some taxa increasing or decreasing during different host life history stages (Bennett et al. 2013; Deveautour et al., 2020; Santos-Gonzalez et al. 2007). Consideration of additional plant species and functional groups is required to determine if the observed trends in network structure can be extended to other grass, forb, and legume species though.

AM fungal spore community seasonal dynamics mirrored seasonal resource partitioning in plant hosts. *B. bracteata*, the spring ephemeral, is active and grows in the early spring, and then flowers by early summer, whereas *(A) gerardii*, the warm season specialist, is most active in the summer and flowers in the early fall. By growing and flowering at different times during the growing season, *(B) bracteata* and *(A) gerardii* (as well as other cool and warm season specialists) partition the growing season and reduce interspecific competition (Weltzin and McPherson 1997; Ford 2010; Doležal et al. 2019). In this study, AM fungal spore community dynamics closely followed this trend, as AM fungal species that were active in the early spring sporulated in early summer (as *(B) bracteata* flowered) and AM fungal communities that were active in the summer demonstrated higher spore diversity when *A. gerardii* flowered (relative to its growth phase). Similar to their plant hosts, this means that AM fungi can also partition the growing season (i.e., cool vs. warm season specialists) and that seasonal differences in AM fungal physiology may contribute to AM fungal coexistence and diversity (Bever et al. 2001). This supports prior work in grassland systems where AM fungal taxa (*Gigaspora gigantea*-cool season and *Acaulospora capsicula*-warm season) displayed distinct seasonal sporulation patterns (Schultz et al. 1999; Pringle and Bever 2002). While not considered in this study, seasonal sporulation patterns may also be influenced by dispersal via aerial propagules (Chaudhary et al. 2020) or animals (Paz et al. 2021), with the contribution of dispersal increasing during times of increased sporulation such as the late Spring and Summer. Additionally, seasonal variation in sporulation may also be affected by environmental conditions such as drought; however, sporulation responses to low rainfall and arid conditions are known to vary so more work is required to understand the effect of drought on AM fungal spore communities (Al-Karaki et al. 2004; Deveautour et al. 2020a). Finally, summer AM fungal network structure did not differ between host-plant species. This suggests that environmental conditions may dominate plant host controls on network structure, and favor greater connectivity and associations between AM fungal symbionts at the end of the growing season (Kaisermann et al. 2015; Bastías et al. 2022). If this effect was due to the low rainfall conditions experienced in summer 2019, this may be evidence of stressful conditions favoring both higher levels of interaction among soil microbes (i.e., the stress gradient hypothesis; David et al. 2018; Hesse et al. 2021) and greater sporulation (Daniels and Skipper 1982). Nevertheless, more work is required to test how water stress influences AM fungal networks across a large set of plant hosts.

In conclusion, AM fungal community assembly displayed strong seasonal trends that differed strongly between host plant species. This work is the first to test how the seasonal dynamics of AM fungal community assembly vary between plant host species, and builds on prior work demonstrating the importance of seasonal variation (Santos-Gonzalez et al. 2007; Deveautour et al. 2020b), changes in AM fungal network structure (Bennett et al. 2013), and plant host species (Bever et al. 1996; Wilson and Hartnett 1998; Koziol and Bever 2015) contributions to AM fungal community assembly. By observing how the seasonal trajectories of AM fungal spore communities varied between host plant species, we demonstrated how between species associations (i.e., biological filters) influence the ongoing seasonal dynamics that determine AM fungal community assembly. Future work should identify how seasonal trends in AM fungal community assembly vary between plant functional groups, environmental conditions, and disturbance regimes. Because temporal dynamics are an important determinant of community assembly, consideration of the processes that shape microbial community assembly over time can help us better understand soil microbial roles in above- and belowground ecosystems.

## Data Availability

Data are available through Dryad: https://datadryad.org/stash/share/F5PXRIoGLDkS33PwFGxlIaYsGFS99ktmxr6Cwj1wTF0.
